# Assessment of peste des petits ruminant’s vaccine wastage along the vaccine supply chain in Mali

**DOI:** 10.3389/fvets.2025.1635447

**Published:** 2025-12-15

**Authors:** Guy Sidwatta Ilboudo, Ahmadou Sow, Cheick Abou Kounta Sidibé, Lokmane Ouedraogo, Theodore Knight-Jones, Cheick Oumar Fomba, Michel Dione

**Affiliations:** 1 International Livestock Research Institute, Ouagadougou, Burkina Faso; 2 International Livestock Research Institute, Bamako, Mali; 3 Central Veterinary Laboratory, Bamako, Mali; 4 Ministry of Economy and Finances, Ouagadougou, Burkina Faso; 5 International Livestock Research Institute, Addis Ababa, Ethiopia; 6 Directorate of Veterinary Services, Bamako, Mali

**Keywords:** sheep, goats, immunization, vaccine loss, control and eradication, vaccine value supply chain

## Abstract

This study was carried out in six regions of Mali to assess peste des petits ruminants (PPR) vaccine wastage along the vaccine supply chain during the vaccination campaign of 2023. Multi-stage stratified sampling was used to select 107 actors involved in the vaccine supply chain, including private veterinarians (*n* = 75), public vaccinators (*n* = 19), regional veterinary officers (*n* = 12), and one senior veterinary officer at the central level. Vaccine actors operated across the dominant small ruminants (SR) production systems in Mali (pastoral, agropastoral, and peri-urban). The World Health Organization field guidelines for monitoring and reducing vaccine wastage was used as a basis for this study. Results show that a quarter of vaccine doses were wasted. The vaccine wastage rate was 24.3% in public sector and 25.4% in private sector with approximately 90% of wastage occurring at the final stage of delivery (veterinary posts, or private veterinarians). At upstream points in the vaccine supply chain, wastage was low in both private and public distribution channels (less than 1%). No statistically significant difference was observed between the public and private sectors in vaccine wastage rates across the different stages of the vaccine distribution chain. The biggest cause of wastage was vaccine being discarded due to denaturation (46.0 and 32.4%, respectively, for the private and public actors), as doses were not used within the short (1 h) time window between reconstitution in the field and use. Also wastage was high due to improper injection (33.8 and 45.6% in public and private sectors), vial breakage (11.2 and 11.1%), and reconstitution errors (7.4 and 8.8%). The massive loss due to denaturation highlights the need for building stronger cold chains along the vaccine supply chain. In hard to reach areas, where cold chain failure is more likely, a vaccine able to remain potent for a longer period before and after reconstitution such as thermotolerant vaccines would add value. Furthermore, capacity of field vaccinators should be enhanced through trainings on best practices regarding vaccination.

## 1 Introduction

Mali is a large country with diverse landscapes, ranging from northern deserts to southern savannas and forests. Spanning 1,240,192 square kilometers, it ranks as the eighth-largest country on the continent. Livestock herding plays a vital role in the economy, with cattle, sheep, goats, and camels being the primary livestock raised. Livestock contributes substantially to household incomes for at least 80% of the rural population, especially women, and accounts for more than 40% of the Gross Domestic Product (GDP) and three quarters of Mali’s exports (1).

According to the livestock estimations provided by the National Directorate of Animal Production and Industries (DNPIA), on 31^st^ December 2022, 43,913,180 sheep and goats were reared in Mali (2). Unfortunately, productivity in the small ruminants (SRs) sector remains low for several reasons, including high disease burden, high lamb/kid mortality, low growth rates, poor nutritional status and absence of long-term breeding programs, resulting in infertility and long lambing and kidding intervals. Added to that, the inadequate institutional capacity strengthening among actors, plus poor links between producers and markets, make the performance of the SR value chain sub-optimal (3, 4).

One of the most devastating diseases affecting SRs is Peste des Petits Ruminants (PPR). This highly contagious viral disease is caused by a morbillivirus closely related to the rinderpest virus. It primarily affects goats and sheep, as well as wild small ruminants and camelids. PPR was first reported in Côte d’Ivoire in 1942 (5). According to a 2013 serological survey conducted across the administrative regions of Mali (excluding Kidal), the overall individual seroprevalence of PPR was 42.6%. However, prevalence rates varied significantly between regions, ranging from 5.5% in Gao to 55.6% in Koulikoro (6).

The global community is increasingly aware of the high economic burden and devastating consequences of PPR. Although there is no cure, the disease can be effectively prevented through vaccination. Effective live attenuated PPR virus vaccines are widely available. The two most commonly used strains, Nigeria/75/1 and Sungri/96, have demonstrated, in experimental settings, robust protection against all known PPRV lineages (7). The Nigeria/75/1 vaccine has also been proven to provide such complete cross-lineage protection in field use in a large number of countries (8).

Hence, in 2015 the PPR Global Eradication Program (PPR-GEP) was launched under the lead of the World Organization for Animal Health (WOAH) and the Food and Agriculture Organization of the United Nations (FAO) PPR secretariat. The objective of the PPR-GEP is to eradicate PPR by 2030, reinforce veterinary services and reduce the impacts of other major infectious diseases of SRs. This will then contribute to fighting rural poverty, ensuring food security and nutrition and strengthening resilience, national economies and achieving Sustainable Development Goals (SDGs). The PPR-GEP recommends mass vaccination for 2–3 rounds to achieve at least 80% coverage of the sheep and goat population above the age of 3 months (9).

Like several countries in Africa, Mali has been implementing its PPR control and eradication strategy since 2017 with mass vaccination as its main component. Despite the efforts made, vaccine coverage rate remains low, less than 10% (10).

Vaccination in Mali is implemented through a public-private partnership (PPP). The 1990s marked the era of “the privatization of veterinary services,” leading to the gradual disengagement of the government from productive and commercial activities and the government is now refocusing on public service (11). Since then, the country has embarked on a policy of partial cost recovery of vaccination by farmers. Exceptions occur in areas requiring humanitarian support due to environmental disasters or conflict, when a vaccine is further subsidized or even provided free of charge to livestock farmers by development organizations. Twenty years later, the animal health situation in Mali is still fragile. The low vaccination coverage rates for all livestock diseases and the limited networks of private veterinarians are indicators of the inadequate performance of veterinary services. In Mali, the “Ovipeste” vaccine (Nigeria/75/1), produced by the Central Veterinary Laboratory (LCV) in Mali (12) is used. The vaccine is mostly packaged and distributed in multidose vials 100 doses and requires strict cold storage throughout until use. The lack of proper cold chain infrastructure is a major cause of failure in most vaccination programs, especially in countries with very high temperatures like Mali (13). It is often impossible to keep vaccines cold when travelling long distances in rural areas and lack or failures of electricity. Without strong vaccine monitoring and conservation along the supply chain, there is high risk of vaccine wastage, translating to economic losses and inefficiencies in vaccination. In 2005, the WHO estimated that approximately half of the vaccines produced globally are wasted and therefore recommended that countries strengthen local vaccine wastage monitoring (14).

Few studies attempt to estimate the vaccine wastage rate in livestock in sub-saharan Africa because of variability in production systems and challenges in data availability. In Mali, no study has been conducted to estimate vaccine wastage rate recorded during annual livestock mass vaccination campaigns. This is important to inform vaccine actors how to optimize vaccination campaigns by limiting vaccine wastage. It is also recommended that the vaccine wastage rate be evaluated at the same time as the vaccination coverage rate for a better appreciation of the wastage rate (14).

This present study was therefore conducted to estimate the wastage of PPR vaccine along the supply chain for the 2023 vaccination campaign, for improved control of PPR and other SR diseases.

## 2 Methods

### 2.1 PPR vaccination framework

**Figure 1:**
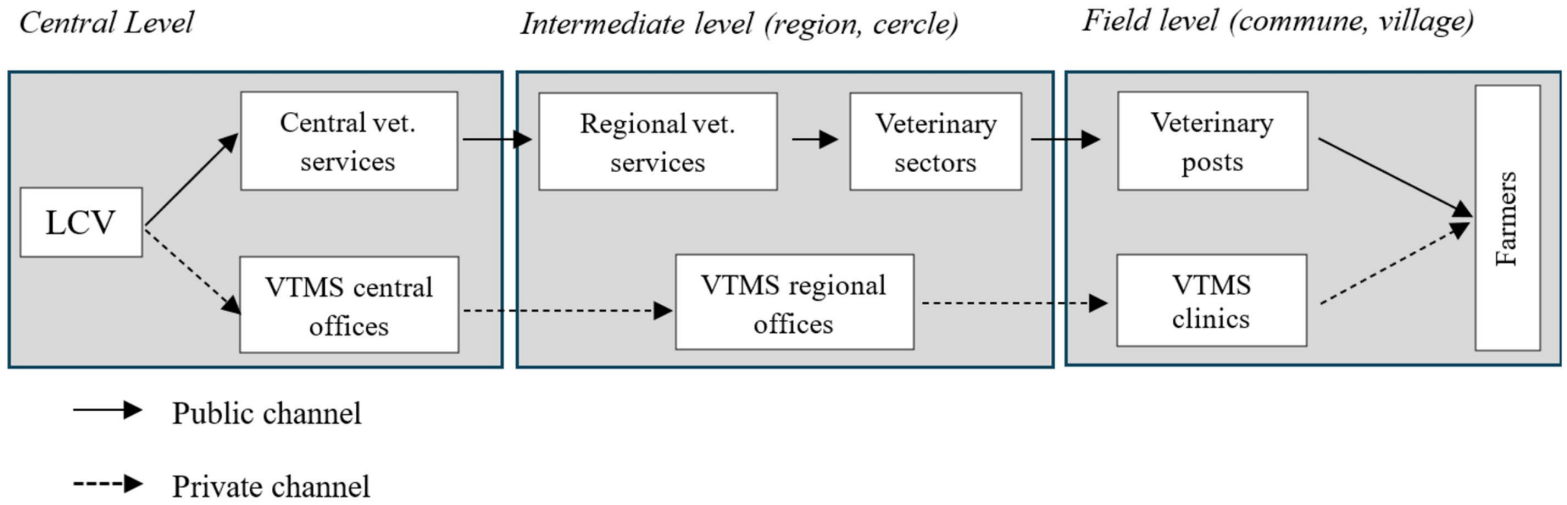
Mapping of PPR vaccine value chains in Mali.

In Mali, the implementation of mass vaccination campaigns against PPR is carried out within the framework of a PPP (15–17). The distribution of the vaccine is done through public and private channels from the central level to the field level. In the public channel, once produced at the Central Veterinary Laboratory (*Laboratoire central vétérinaire –* LCV), the vaccine is stored at the central veterinary services (*Direction nationale des services vétérinaires –* DNSV), then successively shipped to the regional veterinary services (*Directions régionales des services vétérinaires –* DRSV), the departmental veterinary services called Veterinary sector (VS), and the communal veterinary services called veterinary posts (VP). In the private channel, the vaccine is stored in the private veterinarians (*Vétérinaire titulaire du mandat sanitaire* – VTMS) central offices and then shipped successively to the VTMS regional offices and the VTMS veterinary clinics. Vaccination in the field is carried out by the teams of veterinary posts and veterinary clinics (Figure 1). Regarding the vaccination campaign of 2023, the private and public sectors performed 68 and 32% of PPR vaccination, respectively (4).

### 2.2 Study area and sampling

A multi-stage stratified sampling was carried out to select the participants considering the type of SR production, the locality, and the types of actors. Three levels of stratification were then applied.

The first level corresponded to the type of production system, including pastoral, agropastoral, and peri urban. All types of production systems were included in the sample.

The second level of stratification was based on the geographic area, aligned with the country’s administrative divisions, namely regions, cercles, communes, and villages. However, due to the complexity of actor distribution across these levels, stratification was limited to the regional level. The country comprises 20 regions, categorized by production system: 12 in pastoral zones, 6 in agro-pastoral zones, and 2 in peri-urban zones. Within each SR production system, regions were selected purposively, with accessibility and security constraints taken into account. Consequently, in the pastoral zone, the Mopti and Tombouctou regions were selected. In the agro-pastoral zone, the regions of Sikasso, Ségou, and Koutiala were selected. For the peri-urban zone, the Koulikoro region was selected.

The third level of stratification was based on the categories of actors involved in the vaccination campaign, including representatives from the DNSV, DRSV, VS, VP, and VTMS. In each region, the single DRSV representative was selected, and in each cercle, one VS representative was randomly chosen. Across the selected regions, there were 78 VTMS and 40 VP, making a total of 118 vaccinators. All vaccinators who consented were included in the study, bringing the total sample size to 139 participants (Table 1).

**Table 1:** Distribution of the participants to interview over the regions and levels.

Region/level	DNSV	DRSV	VS	VP	VTMS	Total
Central level	1	n/a	n/a	n/a	2	3
VTMS regional offices	n/a		n/a	n/a	6	6
Mopti	n/a	1	1	14	10	19
Tombouctou	n/a	1	1	15	2	11
Sikasso	n/a	1	1	1	13	14
Ségou	n/a	1	1	6	22	26
Koutiala	n/a	1	1	1	9	10
Koulikoro	n/a	1	1	3	22	24
Total	1	6	6	40	86	139

### 2.3 Vaccine wastage rate assessment framework

Vaccine wastage rate can be estimated in several ways but necessarily takes into account the cause of the wastage, including vial breakage, cold chain failure, vaccine disappearance (theft, misplacement, etc.), expiration, missing vials, denaturation and wastage during reconstitution and injection (14, 18–23). Vial breakage can occur throughout the distribution chain, mainly by accident, because vials are made of glass, in particular to preserve the integrity of the albumin used for the stabilization of the vaccine (23–26). The cold chain failure occurs when the vaccine is stored at a temperature outside the recommended range of +2 to +8 degrees Celsius for the case of the thermolabile PPR vaccine (26, 27). This can occur in particular by heat, considering the hot climatic conditions or by freezing (12, 24, 28). The missing vials in the storage boxes are often noted during the inventory after the vaccine has been delivered (14, 29, 30). Wastage during the reconstitution of the vaccine in the field is due either to improper handling by the vaccinator leading to improper dilution, inadequate shaking of vaccines, incomplete aspiration of reconstitution vials, spillage or leakage during reconstitution, a lack of vacuum in the vial containing the vaccine that should be discarded or a manufacturing defect such as a defective cap (31, 32). Wastage during injection corresponds to wastage during removing air of the syringe before injection, injection into a vacuum when the animal is not well restrained or when the vaccinator is not experienced, or contamination of the vial of the already reconstituted vaccine (14, 18, 22, 27). Denaturation of the vaccine corresponds to the loss of its integrity and quality beyond a certain period of time after reconstitution with the diluent made of saline solution, after which these vaccines must be discarded irrespective of the doses used in the vial (14, 33). For example, the thermolabile PPR vaccine produced by the LCV and used for the vaccination campaign must be used within 1 h (12).

In most settings, vaccine distribution follows administrative divisions, which typically involve multiple levels of the supply chain (14, 21, 34, 35). This multi-tiered system entails different levels of vaccine wastage monitoring, as summarized in Table 2 based of literature review. When estimating the wastage rate, it is also important to consider the links in the vaccine value chain (VVC) because the vaccine is distributed through one or more distribution chains. The VVC is a key component of the success of mass vaccination that ensures that the target population is vaccinated. It mainly considers vaccine transport, storage, and use (34, 36, 37).

**Table 2:** Summary of the potential vaccine wastage causes and the levels of the measurement.

Wastage causes categories	Vaccine value chain links			Vaccine distribution levels		
	Transport	Storage	Usage	Central vet. Service	Regional vet. Services and Veterinary Sectors	Public vaccinators
				VTMS central offices	VTMS regional offices	Private veterinarian
Vial breakage	X	X	X	X	X	X
Cold chain failure	X	X	X	X	X	X
Disappearance of vials	X	X	X	X	X	X
Shelf-life expired		X		X	X	X
Missing vials during the inventory		X		X	X	X
Reconstitution errors			X			X
Improper injection			X			X
Denaturation of the vaccine			X			X

Source: summarized from various sources (14, 18–23).

### 2.4 Wastage assessment methods

Based on the types of wastage causes, the wastage rate is calculated by dividing the sum of the wasted doses by the total number of doses initially received through the following Equation 1 (14, 38).


$$q_{v}=\frac{\sum_{k=1}^{n}i_{k}}{N_{v}}\;$$ (1)

With $q_{v}$ the wastage rate at level v , $i_{k}$ the number of wasted doses due to the cause *k*, *k* the cause of wastage, *n* the number of causes of wastage and $N_{v}$ the total number of doses initially received at level v .

Then the usage rate at the level v , which is the complement to 1 of the wastage rate, is calculated from the Equation 2 (14):


$$p_{v}=1-q_{v}$$ (2)

With $p_{v}$ the vaccine usage rate at the level v.

The combined usage rate of each distribution channel (public, private) is calculated based on the Equation 3:


$$p=\prod_{v=1}^{m}p_{v}=\prod_{v=1}^{m}(1-q_{v})$$ (3)

With m the number of distribution levels for each distribution channel (public, private).

The wastage rate q for each distribution channel is then deducted by Equation 4:


$$q=1-p=1-\prod_{v=1}^{m}(1-q_{v})$$ (4)

Finally, the national wastage rate is calculated by considering the share that each distribution channel represents in the coverage rate at the national level, according to the formula in Equation 5:


$$Q=q^{\text{private}}*\frac{D^{\text{private}}}{D_{\text{total}}}+q^{\text{public}}*\frac{D^{\text{public}}}{D_{\text{total}}}$$ (5)

With Q , the national wastage rate, $q^{\text{private}}$ the private wastage rate, $\frac{D^{\text{private}}}{D_{\text{total}}}$ the percentage of animals vaccinated by the private sector, $q^{\text{public}}$ the public wastage rate et $\frac{D^{\text{public}}}{D_{\text{total}}}$ the percentage of animals vaccinated by the public sector. The percentages of animals vaccinated used for weighting were directly derived from official 2023 campaign data and constitute the national reference for the study year. Consequently, this methodological approach focuses on aggregating sectoral data using fixed and validated weights. A sensitivity analysis of alternative weighting assumptions was therefore not deemed necessary (4).

Wastage rates can be estimated at various intervals, annually, quarterly, monthly, or even daily (14, 29). However, given the structure of PPR vaccination activities in Mali, estimating wastage on a per-campaign basis was considered the most appropriate approach. This corresponded to the 2023 vaccination campaign (12 months).

### 2.5 Data collection and analysis

Data were collected through structured, individual face-to-face interviews conducted between October 23 and November 16, 2024. A team of researchers carried out the interviews using a questionnaire deployed via the Open Data Kit (ODK) application on mobile devices (39). The questionnaire was developed to collect data on socio-economic characteristics and aspects related to the transportation, handling, storage, and field use of the PPR vaccine. Specific questions were included to estimate vaccine wastage rates across all stages of the vaccine distribution chain. Eligible respondents included any staff member aged 18 or older who was involved in PPR vaccine management (procurement, transport, storage, or administration) and who provided verbal consent to participate in the interview.

Data collected were entered into an Excel-based spreadsheet and then cleaned and analyzed using STATA 17.0. All wastage rates were calculated using the set of formulas outlined in the subsection 2.4, considering the levels of distribution of the vaccine (central, intermediate and field), the vaccine value chain links (transport, storage and use) and the categories of wastage causes (vial breakage, cold chain failure, vaccine disappearance, expiration, missing vial, denaturation, wastage during reconstitution and injection).

Given the small group sizes (from 1 to 70) of samples and the non-normal distribution of the data, we reported medians and interquartile ranges (IQR) for quantitative variables. The Mann–Whitney U test was used to compare two groups, while the Kruskal-Wallis test was used to compared more than two groups. For categorical variables, proportions were estimated alongside their 95% confidence intervals (CI) using the Wilson method through the Epitools online platform (40). Group comparisons were performed using Fisher’s exact test. For all tests, statistical significance was set at *p* < 0.05.

The results were presented in tables and graphs.

## 3 Results

### 3.1 Socio-economic characteristics of respondents

The results presented in Table 3 show that most of respondents were male in both private sector (97.3%) and public sector (96.9%). In the private sector, most respondents (85.3%) were 55 years or older, whereas in the public sector, the largest age group was 35–54 years old (50%). Furthermore, all private sector respondents had a university level education, while most of the public actors had a secondary education level (56.3%). The primary sources for vaccine procurement also differed, with VTMS regional offices being the most important for the private sector (69.3%) and DRSV for the public sector (75%).

**Table 3:** Various socio-economic information on respondents.

Items	Frequency (percentage; 95% CI)	
	Private actors (*n* = 75)	Public actors (*n* = 32)
Gender		
Male	73 (97.3; 90.8–99.3)	31 (96.9; 84.3–99.4)
Female	2 (2.7; 0.7–9.2)	1 (3.1; 0.6–15.7)
Age category		
18–34	3 (4.0; 1.4–11.1)	10 (31.3; 18.0–48.6)
35–54	8 (10.7; 5.5–19.7)	16 (50.0; 33.6–66.4)
55 and more	64 (85.3; 75.6–91.6)	6 (18.8; 8.9–35.3)
Level of education		
Secondary	0 (0.0; 0.0–4.9)	18 (56.3; 39.3–71.8)
University	75 (100; 95.1–100)	14 (43.8; 28.2–60.7)
Source of vaccine procurement		
VTMS central office	9 (12.0; 6.4–21.3)	0 (0.0; 0.0–10.7)
VTMS regional office	52 (69.3; 58.2–78.6)	0 (0.0; 0.0–10.7)
DNSV	0 (0.0; 0.0–4.9)	5 (15.6; 6.9–31.8)
DRSV	3 (4.0; 1.4–11.1)	24 (75.0; 57.9–86.7)
LCV	3 (4.0; 1.4–11.1)	3 (9.4; 3.2–24.2)
VTMS	8 (10.7; 5.5–19.7)	0 (0.0; 0.0–10.7)
Equipment used to maintain the cold chain during transport		
Vaccine carrier	26 (34.7; 24.9–45.9)	17 (53.1; 36.4–69.1)
Cooler	49 (65.3; 54.1–75.1)	15 (46.9; 30.9–63.6)

### 3.2 Vaccine transport

Vaccine transportation involves moving the vaccines from the supply source to a storage facility, either prior to distribution by other supply chain actors or directly before administration by field personnel. Coolers and vaccine carriers were the most important equipment used to maintain the cold chain during the transport for 65.5% of respondent in the private sector and 53.1% in the public sector (Table 3). The median procurement travel distances were 11, 87, 45, 55, 380, 11, and 401 km for DNSV, VTMS, VP, VS, VTMS regional offices, VTMS central office, and DRSV, respectively. The median duration per procurement was 1.6, 2, 2, 1.0, 4, 0.75, and 5.1 h, respectively. The median transport cost of ice per procurement was USD 0.6, 0.6, 0.8, 1.6, 2.9 and 1,3 for DNSV, VTMS, VP, VS, VTMS regional offices, VTMS central office, and DRSV, respectively. This corresponds to a total ice campaign cost of USD 22.4, 2.6, 2.4, 1.9, 24.7, 36.6 and 1.2, respectively, (Table 4).

**Table 4:** Median (IQR) of various statistics related to PPR vaccine procurement in Mali.

Statistics	VTMS	VP	VS	VTMS regional offices	VTMS central office	DRSV	DNSV	*p*- value
Number of procurements	4 (3–7)	3 (2–4.5)	4 (1–6)	15 (14–25)	9 (4–14)	2 (1–3)	17	*p* < 0.01*
Procurement travel distance (km)	87 (47–150)	45 (40–531)	55 (0.01–120)	380 (160–407)	11 (8–15)	401 (234–655)	11	*p* < 0.01 *
Duration per procurement (h)	2 (1–4)	2 (1–4)	1.0 (0.03–2)	4 (3–5)	0.75 (0.5–1)	5.1 (1–7)	1.6	0.09
Ice cost per vaccine procurement (USD)	0.6 (0.3–0.9)	0.8 (0.3–0.9)	0.6 (0.3–0.8)	1.6 (0.6–1.6)	2.9 (0.9–4.9)	0.7 (0.6–1.1)	1.3	0.32
Ice cost for vaccine per campaign (USD)	2.6 (1.6–4.9)	2.4 (1.1–4.0)	1.9 (0.8–3.4)	24.7 (9.2–41.2)	36.6 (3.9–69.3)	1.2 (0.6–1.9)	22.4	*p* < 0.01*
*n*	70	16	6	3	2	6	1	

1 USD = 606.06 XOF, March 2025. *p*-values correspond to Kruskal-Wallis tests comparing groups for each indicator.

A significant *p*-value was observed for the number of vaccines procured, the distance traveled for procurement, and the ice cost for vaccine transportation.

### 3.3 Vaccine storage

The vaccine is stored at the central level (National veterinary services and VTMS central office), intermediate level (DRSV, VTMS regional offices and VS), and field level (VTMS et VP). Table 5 show that the two main primary sources of electricity for the vaccine storage were the national electricity provider called *Énergie du Mali (EDM)* (42.7 and 53.1% for private actors and public actors, respectively) and solar energy (37.3 and 37.5%). In both private and public sectors, most of the actors (64 and 62.5%) do not have a secondary source of electricity for vaccine storage. No significant difference was observed between the two groups regarding the primary and secondary sources of electricity.

**Table 5:** Primary and secondary sources of electricity for PPR vaccine storage used by private and public actors in Mali.

Items	Frequency (percentage; 95% CI)		*p*-value
	Private actors (*n* = 75)	Public actors (*n* = 32)	
Primary sources of electricity for vaccine storage			
National electricity network (EDM)	32 (42.7; 32.1–53.9)	17 (53.1; 36.4–69.1)	
Solar energy	28 (37.3; 27.3–48.6)	12 (37.5; 22.9–54.7)	0.57
Private providers	9 (12.0; 6.4–21.3)	1 (3.1; 0.6–15.7)	
None, I use ice everyday	4 (5.3; 2.1–12.9)	2 (6.2; 1.7–20.1)	
Others	2 (2.7; 0.7–9.2)	0 (0.0; 0.0–10.7)	
Secondary sources of electricity for vaccine storage			
National electricity network (EDM)	2 (2.7; 0.7–9.2)	2 (6.2; 1.7–20.1)	0.75
Solar energy	16 (21.3; 13.6–31.9)	7 (21.9; 11.0–38.8)	
Private providers	3 (4.0; 1.4–11.1)	2 (6.2; 1.7–20.1)	
Others	6 (8.0; 3.7–16.4)	1 (3.1; 0.6–15.7)	
None	48 (64.0; 52.7–73.9)	20 (62.5)	

*p*-values correspond to Exact fisher tests comparing groups for each indicator. “Others” includes generator, fuel, and gas.

### 3.4 Vaccine use

Vaccine is used in the field by the private veterinarians and the public vaccinators (VP). Table 6 shows that the median number of vaccinated animals per campaign per vaccinator was 5,890 and 9,000, respectively, for the private and the public vaccinators.

**Table 6:** Median (IQR) various statistics related to field use of PPR vaccine in Mali.

Items	VTMS (*n* = 70)	VP (*n* = 19)	*p*-value
No vaccinated animals per campaign per vaccinator	5,890 (2,744-13,735)	9,000 (4,700-25,800)	0.54
Shortest distance to travel for vaccination (km)	1 (0.5–3)	1 (0.1–3)	0.48
Time to travel the shortest distance (minutes)	10 (5–15)	10 (5–30)	0.57
Longest distance to vaccinate (km)	32.5 (27–48)	28 (15–45)	0.39
Time to travel the longest distance (hours)	1.5 (1–2)	1 (1–2.5)	0.70
No villages covered per day	2 (1–3)	2 (1–2)	0.71
No farmers covered per day per vaccinator	10 (5–20)	10 (5–20)	0.93
No animals covered with vial of 100 doses	90 (80–95)	95 (90–97)	0.07
No farmers covered with vial of 100 doses	3 (2–5)	3 (2–4)	0.63
Cost of ice per day (USD)	0.5 (0.3–0.6)	0.6 (0.5–1.6)	0.08
Cost of ice per campaign (USD)	11.8 (6.6–23.9)	14.8 (4.6–46.2)	0.91

*p*-values correspond to Mann–Whitney *U* test comparing groups for each indicator.

The shortest median distance travelled to vaccinate was 1 km for both, corresponding to a time taken of 10 min. The longest median distance travelled to vaccinate was 32.5 and 28 km for private and public vaccinators respectively, corresponding to a time taken of 1.5 and 1 h.

In one day of vaccination, both private and public vaccinators cover on 2 villages as well as 10 farmers.

With a vial of 100 doses, they vaccinate a median number of 90 and 95 animals in the private and public sector, respectively. This represents 3 farmers per vial in both the private and public sectors.

The cost of ice per vaccination day in the field was estimated at USD 0.5 and 0.6 in the private and public sectors, respectively. This makes a total cost per campaign of USD 11.8 and 14.8 of ice used in the field, respectively, in the private and public sectors. No significant difference was observed between the two groups regarding these statistics (Table 6).

### 3.5 Vaccine wastage rate

#### 3.5.1 Respondent statements on the vaccine wastage causes

During vaccine transportation, 34.7% of respondents in the private sector and 37.5% in the public sector reported vial breakage. Only a small proportion reported wastage due to cold chain failures (4.0 and 3.1%) or vaccine disappearance (2.7 and 0.0%). During storage, few proportions also reported wastage due to vial breakage (16.0 and 9.4%), cold chain failure (5.3 and 9.4), vaccine disappearance (1.3 and 0.0%), vaccine expiration (4.0 and 18.8%) or missing vial (6.7 and 0.0%). In the field, the main causes of vaccine wastage during use were vaccine denaturation (reported by 92.9 and 89.9% of respondents in the public and private sectors, respectively), improper injection (88.6 and 100%), errors during reconstitution (28.6 and 36.8%), and vial breakage (20.0 and 36.8%). No statistically significant differences were observed between the two sectors regarding the causes of vaccine wastage across the different stages of distribution, except the vaccine expiration during the storage mostly observed in the public sector (Table 7).

**Table 7:** Reported causes of PPR vaccine wastage during transportation, storage and usage in the private and public sectors in Mali.

Items	Frequency (percentage; 95% CI)		*p*-value
	Private sector	Public sector	
During vaccine transportation, have you recorded vial breakage? (*n* = 75; 32)			
Yes	26 (34.7; 24.9–45.9)	12 (37.5; 22.9–54.7)	0.82
No	49 (65.3; 54.1–75.1)	20 (62.5; 45.3–77.1)	
During vaccine transportation, have you recorded cold chain failure? (*n* = 75; 32)			
Yes	3 (4.0; 1.4–11.1)	1 (3.1; 0.6–15.7)	1.00
No	72 (96.0; 88.9–98.6)	31 (96.9; 84.3–99.4)	
During vaccine transportation, have you recorded vaccine disappearance? (*n* = 75; 32)			
Yes	2 (2.7; 0.7–9.2)	0 (0.0; 0.0–10.7)	1.00
No	73 (97.3; 90.8–99.3)	32 (100; 89.3–100)	
During vaccine storage, have you recorded vial breakage? (*n* = 75; 32)			
Yes	12 (16.0; 9.4–25.9)	3 (9.4; 3.2–24.2)	0.54
No	63 (84.0; 74.1–90.6)	29 (90.6; 75.8–96.8)	
During vaccine storage, have you recorded cold chain failure? (*n* = 75; 32)			
Yes	4 (5.3; 2.1–12.9)	3 (9.4; 3.2–24.2)	0.42
No	71 (94.7; 87.1–97.9)	29 (90.6; 75.8–96.8)	
During vaccine storage, have you recorded vaccine disappearance? (*n* = 75; 32)			
Yes	1 (1.3; 0.2–7.2)	0 (0.0; 0.0–10.7)	1.00
No	74 (98.7; 92.8–99.8)	32 (100; 89.3–100)	
During vaccine storage, have you recorded vaccine expiration? (*n* = 75; 32)			
Yes	3 (4.0; 1.4–11.1)	6 (18.8; 8.9–35.3)	0.02*
No	72 (96.0; 88.9–98.6)	26 (81.2; 64.7–91.1)	
During vaccine storage, have you recorded missing vial? (*n* = 75; 32)			
Yes	5 (6.7; 2.9–14.7)	0 (0.0; 0.0–10.7)	0.31
No	70 (93.3; 85.3–97.1)	32 (100; 89.3–100)	
During vaccine usage, have you recorded vial breakage? (*n* = 70; 19)			
Yes	14 (20.0; 12.3–30.8)	7 (36.8; 19.1–58.7)	0.13
No	56 (80.0; 69.2–87.7)	12 (63.2; 41.0–80.9)	
During vaccine usage, have you recorded cold chain failure? (*n* = 70; 19)			
Yes	1 (1.4; 0.3–7.7)	1 (5.3; 0.9–24.6)	0.38
No	69 (98.6; 92.3–99.7)	18 (94.7; 75.4–99.1)	
During vaccine usage, have sample the vaccine for awareness raising session? (*n* = 70; 19)			
Yes	2 (2.9; 0.8–9.8)	0 (0.0; 0.0–16.8)	1.0
No	68 (97.1; 90.2–99.2)	19 (100; 83.2–100)	
During vaccine usage, have you recorded vaccine wastage by reconstituting the vaccine? (*n* = 70; 19)			
Yes	20 (28.6; 19.3–40.1)	7 (36.8; 19.1–58.7)	0.56
No	50 (71.4; 59.9–80.7)	12 (63.2; 41.0–80.9)	
During vaccine usage, have you recorded denaturation of the vaccine? (*n* = 70; 19)			
Yes	65 (92.9; 84.3–96.9)	17 (89.5; 68.6–97.1)	0.63
No	5 (7.1; 3.1–15.7)	2 (10.5; 2.9–31.4)	
During vaccine usage, have you recorded wastage during the injection? (*n* = 70; 19)			
Yes	62 (88.6; 79.0–94.1)	19 (100; 83.2–100)	0.19
No	8 (11.4; 5.9–21.0)	0 (0.0; 0.0–16.8)	

*p*-values correspond to Exact Fisher tests comparing groups for each indicator. *p-value statistically significant (<0.05).

#### 3.5.2 Estimated wastage rate description

The media wastage rate is estimated at 0.39, 0.08, 0.0, 0.45, 0.0, 24.0 and 25.0%, respectively at the DNSV, VTMS central offices, DRSV, VTMS regional offices, VS, VP and VTMS levels. Thus, important wastage rates were observed at the field level in both public and private sector. No statistically significant differences were observed between the public and private sectors in vaccine wastage rates across the different stages of the distribution chain. Combining the wastage rate of all vaccine distribution levels, the wastage rate for the private sector and public sector is estimated at 25.4 and 24.3% respectively, making an overall wastage rate of 25.0% at national level (Table 8).

**Table 8:** PPR vaccine wastage rates along the vaccine value chains in Mali.

Level	n	Median (IQR)	Stage	p-value
DNSV	1	0.39	Central	0.22
VTMS central offices	2	0.08 (0–0.16)		
DRSV	6	0.0 (0–0)	Intermediate	0.38
VTMS regional offices	3	0.45 (0–4)		
VS	6	0.0 (0–3)		
VP	15	24.0 (14.7–34)	Field	0.71
VTMS	70	25.0 (15–40)		
Private sector	1	25.4	National	n/a
Public sector	1	24.3		n/a
National	1	25.0		n/a

*p*-values correspond to Kruskal–Wallis tests comparing groups within each stage.

**Figure 2:**
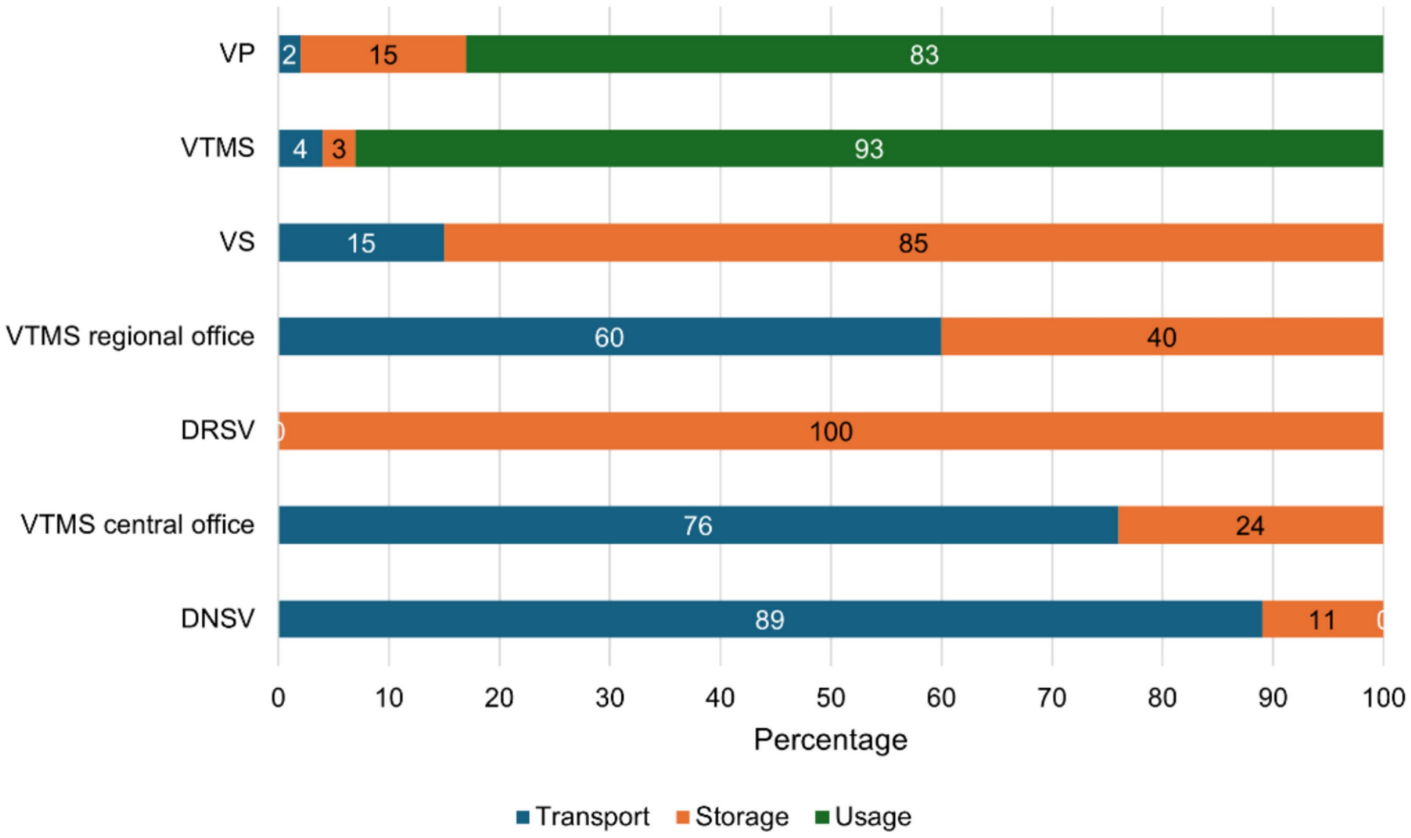
Distribution of place of PPR vaccine wastage over the vaccine value chain levels in Mali.

As shown in Figure 2, most wastage occurred during transport for DNSV, central and regional VTMS offices (89, 76 and 60%), during storage at the DRSV and VS levels (100 and 85%) and during use at the VTMS and VP levels (93 and 83%).

**Figure 3:**
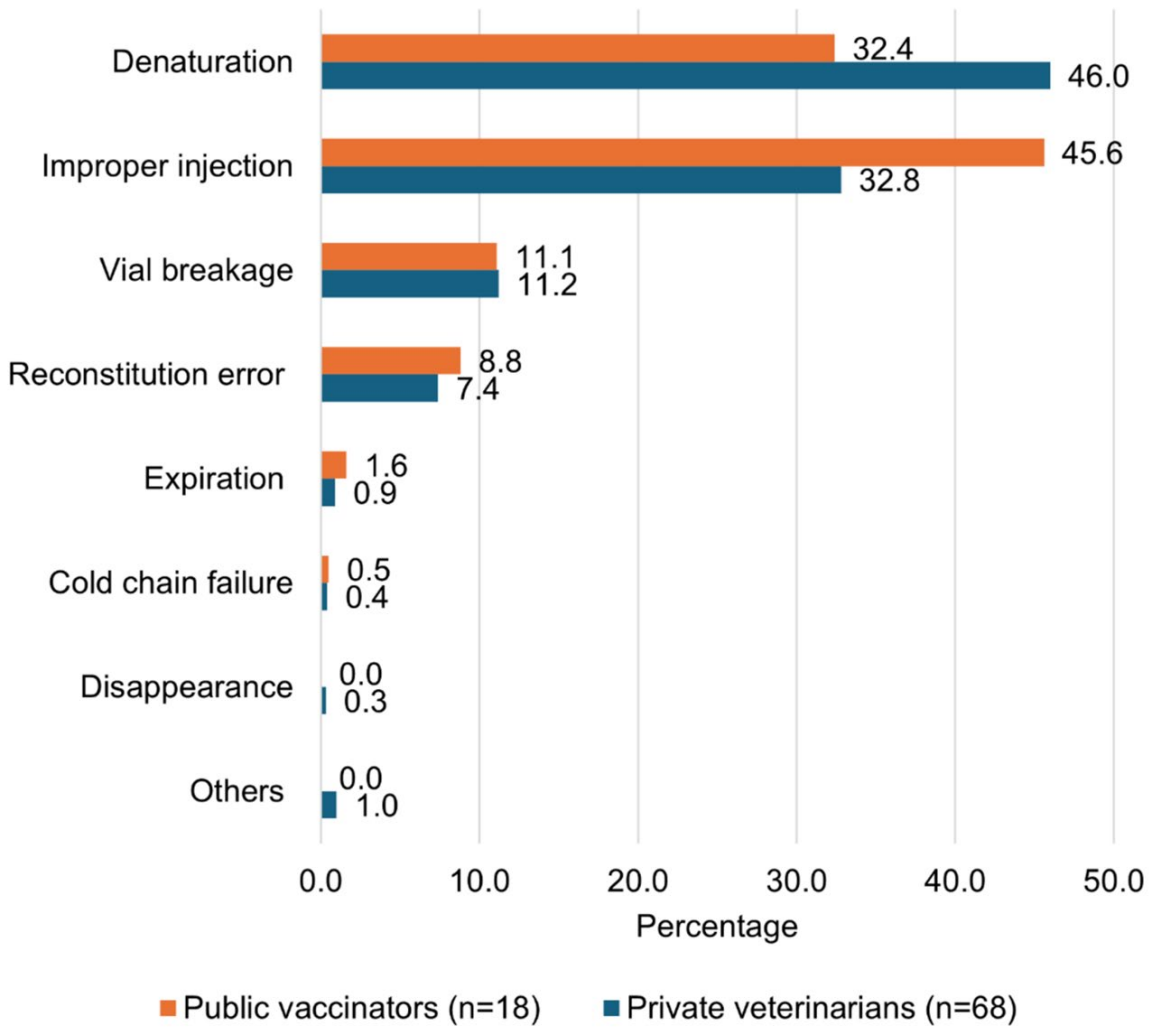
Distribution of PPR wastage causes over the private and public vaccinators at field level in Mali.

**Figure 4:**
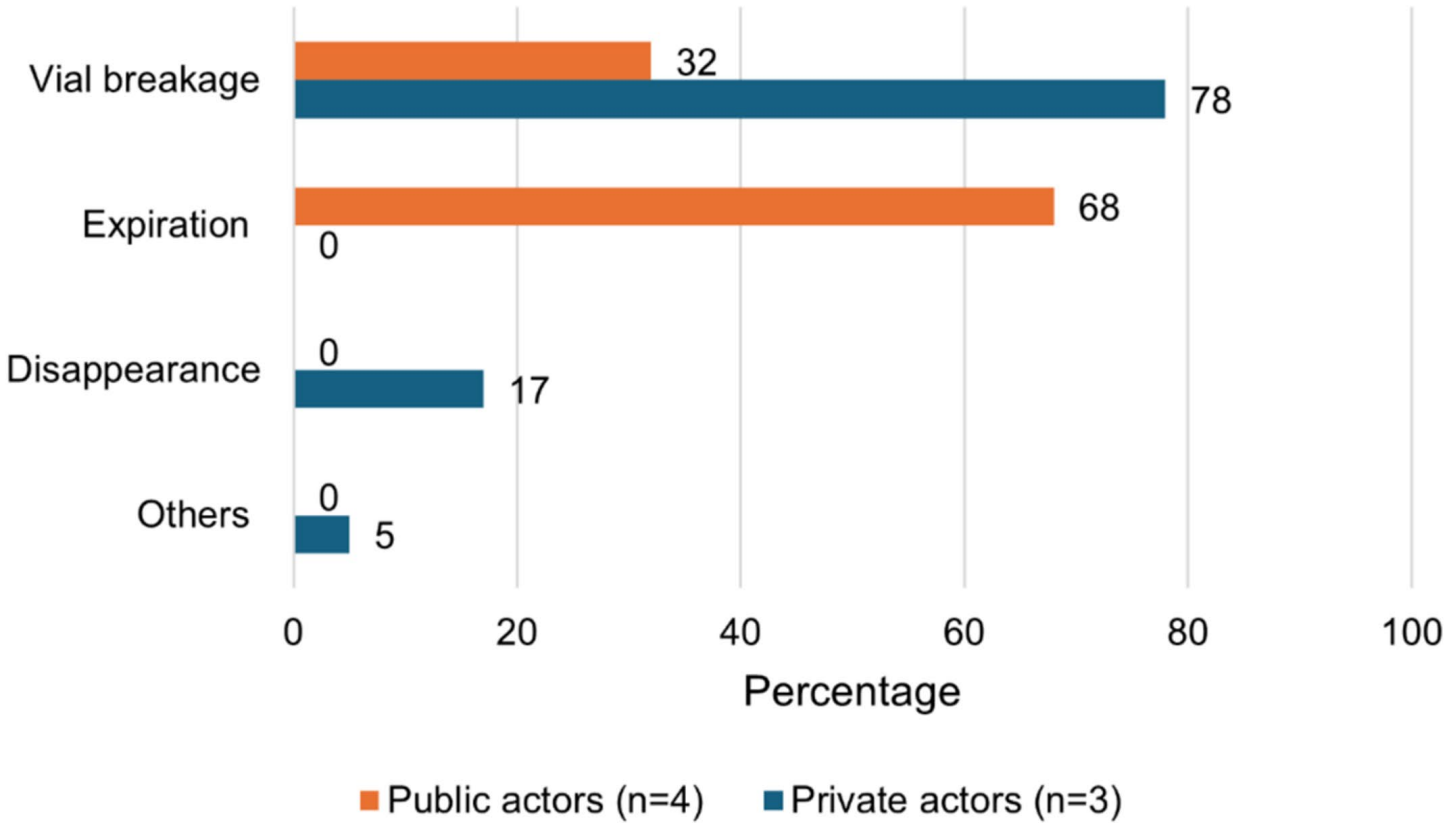
Distribution of PPR wastage causes over the private and public actors at intermediate level in Mali.

The top four causes of wastage were vaccine denaturation (46.0 and 32.4% in the private and public actors), improper injection (32.8 and 45.6%), vial breakage (11.2 and 11.1%), and reconstitution errors (7.4 and 8.8%). The cold chain failure was estimated to represent only 0.4 and 0.5% of the wastage rate, respectively, in the private and public sectors (Figure 3). At the intermediate level, wastage is due to vial breakage (32%) and expiration (68%) in the public sector while it is mainly caused by vial breakage (78%) and disappearance (17%) in the private sector (Figure 4).

## 4 Discussion

- Vaccine transport, storage, and use highlights

Vaccines are primarily transported from supply sources to storage or vaccination sites using coolers, although, these containers have limited capacity to effectively maintain the cold chain (26). Depending on the distribution level, vaccines are transported over distances ranging from 11 km to more than 400 km with significant difference for regional actors, due to the considerable separation between regional capitals and Bamako, the national capital. They typically require a minimum of 4 h, while those operating at intermediate or field levels generally take 1–2 h, despite covering shorter distances. This is largely due to factors such as the modes of transportation, primarily motorcycles, and poor road conditions, which can prolong transit time for field-level personnel. Interestingly, the cost of ice used to preserve vaccines during transport is relatively uniform across all distribution levels, ranging from USD 0.6 to 1.9 per trip for most cases. However, given the disparities in vaccine volumes transported, this uniformity suggests a proportionally higher ice cost at the field level. This is likely attributable to longer transport durations and suboptimal transport conditions.

Regarding vaccine storage, only half of stakeholders rely primarily on the electricity supplied by public utilities supplemented by personal solar energy systems. This trend reflects the ongoing energy crisis, with public electricity coverage reaching only 52% nationwide and just 24% in rural areas (41). In particular, central veterinary services that lack functional solar installations refrain from maintaining vaccine stockpiles, to minimize losses caused by cold chain failure during power outages. The absence of secondary sources of energy is also noted by at least two-third of stakeholders posing a risk to vaccine quality, as cold chain failure may occur without being detected, especially in the absence of a temperature monitoring and recording system. This not only jeopardizes vaccination efficacy but also limits the capacity to accurately estimate vaccine wastage rate linked to cold chain failures.

The study highlighted the limited capacity of vaccinators to cover the target populations. In fact, in one day of vaccination, both private and public vaccinators can only cover two villages corresponding to 10 farmers. This might be due to the spread distribution villages and herd in the context of Mali combining to the low logistics of actors (mean of transport, insufficiency of vaccinators and vaccination materiel). Moreover, accessing certain locations can be challenging due to their remoteness and poor road conditions, particularly during the rainy season. The nature of the vaccine is also a major constraint considering the context. Indeed, a 100-dose vaccine vial typically covers only three farmers considering the small-scale SR herds managed by most of farmers. Consequently, due to the limited time window (maximum 1 h) for using vaccines after vial opening, combined with the logistical challenges of assembling animals and the low number of doses required per herd, only a limited number of animals can be vaccinated from a single vial (33, 42). This constraint leads to considerable vaccine wastage, not only due to denaturation after vial opening but also from residual losses during administration. Efforts should focus on strengthening the logistical capacity of field actors to enable them to vaccinate a larger number of animals per day. This would help shorten the duration of vaccination campaigns, which is crucial for the efficient management of limited resources.

- Significant wastage predominantly occurs in the field

The wastage rates are estimated at 24.3 and 25.4% for the public and private sectors, respectively, resulting in a national wastage rate of 25%. The WHO acknowledges that establishing a universally acceptable vaccine wastage threshold is not feasible, as it varies depending on vaccination programs, local contexts, disease profiles, and vaccine types (14, 43). Studies conducted in various regions on comparable freeze-dried vaccines used in human immunization programs have reported a wide range of wastage rates, varying from 5 to 79% (29, 30, 33, 44, 45). However, few studies have addressed vaccine wastage within the field of animal health. In Ethiopia, for instance, the wastage rate of the PPR vaccine was estimated at 22% (18). Regardless, vaccination coverage remains the most critical factor to consider. The WHO recommends factoring in vaccination coverage when evaluating wastage rates. The correlation between vaccine wastage rate and immunization coverage is crucial for determining whether wastage levels are relatively excessive. Both metrics should be analyzed over time rather than at a single time point to identify trends (14). Based on this approach, the estimated wastage rate of 25% observed in the current study is considered notably high, particularly given the low vaccination coverage of only 10% (10).

Our study shows that wastage is much lower at the central and intermediate levels compared to the field level. Indeed, these levels only cover vaccine transport and storage, and the wastage occurs mainly with unopened vials (29, 33, 45, 46). In contrast, wastage at the field level predominantly results from factors such as denaturation, improper injection, vial breakage, and reconstitution errors. These four causes alone account for at least 97% of wastage in both the public and private sectors. Wastage is mainly recorded during vaccine transport, storage, and use, respectively, for actors at the central, intermediate, and field levels. This once again highlights the need to strengthen resource allocation to field actors to improve the implementation of vaccination campaigns.

Denaturation is one of the main factors contributing to vaccine wastage in this study. It can be attributed to the short timeframe for vaccine use after reconstitution (typically 1 h), small flock sizes, and the logistical challenges of assembling animals for vaccination in Mali context (47, 48). Numerous studies have shown that the wastage rate of freeze-dried vaccines is significantly higher than that of liquid vaccines, primarily due to their greater susceptibility to denaturation once opened (29, 33, 49). Unfortunately, only honest actors are likely to adhere to this deadline because a denatured vaccine cannot be differentiated from a normal vaccine at first sight. A vaccinator could easily mislead farmers and continue administering vaccines in order to maximize personal profit. This highlights the importance of vigilance among farmers when using this type of vaccine. Their active participation in vaccine quality control is crucial, especially when dealing with vaccinators who may have limited training. Moreover, many vaccinators shift the burden of vaccine wastage onto farmers by charging them for the full cost of a 100-dose vial, regardless of the number of animals vaccinated. They administer doses to the available animals and discard the remaining volume. This type of wastage often goes unnoticed at the national level. As a result, some vaccinators may report the number of doses paid for rather than the actual number of animals vaccinated, leading to distorted estimates of vaccination coverage. A practical, though not necessarily cheaper, alternative would be to use vials with fewer doses to help minimize such wastage (5, 44). Wastage can also be reduced by organizing sessions with large numbers of animals (grouping herds), so even if the last vial used has doses remaining, it is a small proportion of the doses distributed during the session.

Vaccine wastage during injection is frequently associated with the vaccinator’s level of experience and the adequacy of animal restraint. Despite the high level of education of the respondents, it is essential to provide thorough training to vaccinators, particularly when involving inexperienced vaccinators and community-level personnel with limited technical backgrounds. Similar attention should be given to procedures related to vaccine reconstitution, which can also be a significant source of wastage.

Vial breakages are frequently observed due to inadequate transport conditions, particularly when vaccines are carried in coolers where ice packs and vaccine vials are placed haphazardly, which is common in the context of Mali. This increases the risk of impact between the ice packs and the vials, leading to breakage (26). Additionally, some actors store the vaccine in freezers at −20 °C, as recommended by the manufacturer for long-term storage (up to 2 years) (12). However, the vials often become frozen solid, increasing the risk of damage, especially during removal if handled carelessly.

- Addressing cold chain failures: thermotolerant vaccines as a sustainable solution

Cold chain failures appear to be a minor contributor to vaccine wastage in contrast to the conclusions reported by Michel et al. (13). In the public sector, only 3.1, 9.4, and 5.3% of respondents reported experiencing cold chain failure during transport, storage, and field use, respectively. Similarly, in the private sector, 4.0, 5.3, and 1.4% of respondents reported such failures during the same phases. Moreover, cold chain failures contributed minimally to overall vaccine wastage, accounting for only 0.4 and 0.5% in the private and public sectors, respectively. Although there is an important risk of underestimating the wastage related to cold chain failure (see study limitations in the following section), this wastage deserves to be seriously considered in vaccination programs. As precautionary measure, it is essential to transport vaccines using insulated containers with tight-fitting lids. When lined with ice packs, these containers help maintain the required cold temperatures for both vaccines and diluents during transport and/or temporary storage (26). Moreover, the use of thermotolerant vaccines offers a significant advantage in hot and arid regions such as Mali, where cold chain infrastructure is limited and where recent electricity shortages have further exacerbated storage challenges (13, 50, 51). For instance, the PPR thermotolerant vaccine developed by the International Livestock Research Institute (ILRI), in collaboration with Mali Central veterinary laboratory (LCV) and Hester Biosciences, can be stored or transported at 32.5 degrees Celsius for 9 days (52). Additionally, it remains viable for up to 5 h after reconstitution (53), which greatly reduces wastage related to denaturation, previously accounting for 46.0 and 32.4% of field wastage in the private and public sectors, respectively.

- Study limitations

The limitation of this study is the approach used to estimate the wastage due to cold chain failure. The findings were based on a cross-sectional study relying on self-reported data collected during interviews. Since temperature fluctuations were not directly monitored, cold chain-related wastage may be underestimated, as breaches can occur without the knowledge of those managing the cold chain. This concern is particularly relevant in the current context in Mali, where there are frequent power outages, the lack of backup power sources for most actors, the long distances of vaccine transportation and the use of inadequate vaccine coolers during transport might further compromise cold chain integrity. Besides, Dione et al., have raised concerns about the quality of vaccines delivered to farmers in Mali regarding the low capacity of veterinarians to store the vaccine at required temperatures (48). More rigorous approaches should be used to better estimate the wastage due to cold chain failure by recording the temperatures through all levels of the PPR vaccine distribution over the time. Moreover, implementing a reliable temperature monitoring and recording system is a critical component of PPR control and eradication strategies, even in developed countries. For instance, a temperature monitoring study conducted by Young et al. (26) for a month in India, the vaccine supply system showed significant peaks in temperatures (above 10 °C). A study conducted by Scott and Shannon (54) in the United States on the supply chain of animal health products showed that only a third of the refrigerators of the distributors tested operated within the acceptable temperature range. Among those who claimed to monitor temperatures, some relied solely on subjective assessment, such as noting that the vaccines felt “cool” upon removal, rather than using proper thermometric equipment. Even inside a freezer, temperature variations can occur depending on the specific location where the vaccines are stored (55).

## 5 Conclusion

This study revealed a high vaccine wastage rate during PPR vaccination in Mali, at the same time as low vaccination coverage. Wastage primarily occurs at the field level through denaturation, improper injection, vial breakage, and reconstitution errors. Such wastage poses a significant challenge to the PPR control strategy, which heavily relies on mass vaccination. To mitigate field-level wastage, it is essential to implement several measures: adopting vials that contain fewer vaccine doses per vial, using vaccines with longer stability post-reconstitution, promoting thermotolerant vaccines, enhancing vaccinator training, and improving the organization of vaccination campaigns. Additionally, regular temperature monitoring and recording are crucial for better management cold chain-related wastage.

## Data Availability

The raw data supporting the conclusions of this article will be made available by the authors, without undue reservation.
